# Factors associated with parents’ experiences using a knowledge translation tool for vaccination pain management: a qualitative study

**DOI:** 10.1186/s12913-021-06326-2

**Published:** 2021-04-16

**Authors:** Nicole E. MacKenzie, Perri R. Tutelman, Christine T. Chambers, Jennifer A. Parker, Noni E. MacDonald, C. Meghan McMurtry, Pierre Pluye, Vera Granikov, Anna Taddio, Melanie Barwick, Kathryn A. Birnie, Katelynn E. Boerner

**Affiliations:** 1 Dalhousie University, Department of Psychology and Neuroscience, Halifax, Nova Scotia Canada; 2 IWK Health Centre, Centre for Pediatric Pain Research, Halifax, Nova Scotia Canada; 3 Dalhousie University, Department of Pediatrics, Halifax, Nova Scotia Canada; 4 University of Guelph, Department of Psychology, Guelph, Ontario, Canada and Pediatric Chronic Pain Program, McMaster Children’s Hospital, Hamilton, Ontario Canada; 5 McGill University, Department of Family Medicine, Montreal, Quebec Canada; 6 University of Toronto, Leslie Dan Faculty of Pharmacy, Toronto, Ontario Canada; 7 Child Health Evaluative Sciences Research Institute, The Hospital for Sick Children, Toronto, Ontario Canada; 8 8University of Toronto, Department of Psychiatry, Faculty of Medicine and University of Toronto, Dalla Lana School of Public Health, Toronto, Ontario Canada; 9 University of Calgary, Department of Anesthesiology, Perioperative and Pain Medicine, Calgary, Alberta Canada; 10 BC Children’s Hospital & University of British Columbia, Department of Psychiatry, Vancouver, British Columbia Canada

**Keywords:** Vaccination, Pain management, Pediatric pain, Knowledge translation, Evidence-based practices, Evidence uptake

## Abstract

**Background:**

Vaccination is a common painful procedure for children. Parents’ concern regarding vaccination pain is a significant driver of vaccine hesitancy. Despite the wealth of evidence-based practices available for managing vaccination pain, parents lack knowledge of, and access to, these strategies. Knowledge translation (KT) tools can communicate evidence-based information to parents, however little is known about what factors influence parents’ use of these tools. A two-page, electronic KT tool on psychological, physical, and pharmacological vaccination pain management strategies for children, was shared with parents as part of a larger mixed methods study, using explanatory sequential design, exploring factors related to uptake of this KT tool. The aim of this qualitative study was to understand what influenced parents’ perceptions of the relevance of the KT tool, as well as their decision as to whether to use the tool.

**Methods:**

A qualitative descriptive design was used. A total of 20 parents of children aged 0–17 years (*n* = 19 mothers) reviewed the KT tool ahead of their child’s upcoming vaccination and participated in a semi-structured interview at follow-up. Interviews were recorded, transcribed verbatim, and analyzed with reflexive thematic analysis using an inductive approach.

**Results:**

The analysis generated three interrelated themes which described factors related to parents’ use of the KT tool: (1) Relevance to parents’ needs and circumstances surrounding their child’s vaccination; (2) Alignment with parents’ personal values around, and experiences with, vaccination pain management (e.g., the importance of managing pain); and (3) Support from the clinical environment for implementing evidence-based strategies (e.g., physical clinical environment and quality of interactions with the health care provider).

**Conclusions:**

Several factors were identified as central to parents’ use of the KT tool, including the information itself and the clinical environment. When the tool was perceived as relevant, aligned with parents’ values, and was supported by health care providers, parents were more inclined to use the KT tool to manage their children’s vaccination pain. Future research could explore other factors related to promoting engagement and uptake when creating parent-directed KT tools for a range of health-related contexts.

**Supplementary Information:**

The online version contains supplementary material available at 10.1186/s12913-021-06326-2.

Vaccinations are a common painful procedure in childhood [1]. In Canada, children will receive approximately 20 vaccinations by the time they enter school, as per the national schedule for vaccination [2, 3]. Poorly managed vaccination pain can lead to greater pain and distress during subsequent vaccinations [4], and although most parents report a desire to manage their children’s vaccination pain, many are unaware of the existing evidence and strategies to do this [3]. Furthermore, health care providers (HCPs) often do not discuss vaccination pain management with parents unless the parent raises the topic [3]. While some parents intuitively use helpful strategies to manage their child’s pain (e.g., distraction), a majority of parents unknowingly use methods to manage their child’s pain that have been shown to be ineffective (i.e., reassurance) [5, 6]. Thus, a significant knowledge-to-action gap exists between parents and their access to evidence-based strategies to manage children’s vaccination pain. Making evidence-based strategies for vaccination pain management available to parents is critical as parental concern regarding needle pain has been identified as a significant barrier to children receiving their vaccinations [5]. Parents can be powerful advocates for their child’s pain management [7], however scientific evidence on pain management is often inaccessible to parents [3], both physically and in terms of language.

Knowledge translation (KT) is an iterative process that involves summarizing scientific literature and sharing it with knowledge users in a way that is easy to understand and apply [8]. KT has been used in the field of children’s pain to present evidence to parents and children on vaccination pain management strategies in an effort to close the knowledge-to-action gap [9–12]. KT activities, such as organizational evaluations (e.g., outcome evaluation, quality improvement) [13] and creation of resources and tools for HCPs (e.g., clinical practice guidelines) and parents (e.g., KT tools and other formats for evidence dissemination geared toward parents) [8] play an integral role in the uptake and use of evidence in practice and have been shown to have significant positive impacts on health care and related service provision [14]. In terms of patient-directed efforts, KT tools have also shown positive impacts on patients’ own knowledge about their health care, health behaviours, and communication with their HCP regarding their health care [14].

In an effort to disseminate evidence-based vaccination pain management strategies to parents, a two-page KT tool (see Additional Files), written in plain language, was previously developed to share these strategies with parents. This tool was based on a clinical practice guideline on this subject [15] and communicated the evidence-based recommendations for each developmental age group (i.e., infancy, school age, and adolescents) in plain language. This KT tool was developed by a multidisciplinary research team in collaboration with Immunize Canada [16], an organization which promotes understanding and use of vaccines. The tool presented pharmacological (e.g., topical anesthetic), psychological (e.g., distraction), and physical strategies (e.g., positioning of the child) for managing children’s vaccination pain. While pain management KT tools similar to this one have been developed for parents and general uptake studied in the past [17], the exploration of specific factors predicting the benefits, including use of the evidence, resulting from parents’ use of KT tools has not been conducted.

In order to address this gap, we carried out a mixed methods study, using an explanatory sequential design [18] to better understand factors influencing parents’ use of KT tools to manage children’s pain. The overall study was guided by The Information Assessment Method (IAM), which is based on a theoretical framework that proposes four factors central to the effectiveness of KT tools, and uptake of evidence-based information: situational relevance and cognitive impacts of the information (aligned with beliefs), intentions to use information, and anticipated benefits [19, 20].

This overall study consisted of two phases: (1) An initial quantitative phase to determine factors associated with uptake of a KT tool; (2) A qualitative phase to further explore the quantitative study results in-depth. The initial quantitative phase presented parents with the aforementioned KT tool and consisted of an online survey that examined parents’ opinions of the KT tool, and plans to use it, prior to their child’s upcoming vaccination appointment. Approximately six months following their child’s vaccination, parents were invited to complete a follow-up survey to report on their actual use and perceptions of the KT tool, as well as their perceptions of the tool, having used it or not. 128 parents participated at follow-up and it was found that the relevance of the KT tool predicted plans to use it, and confidence in use of the tool predicted actual use of it at the vaccination appointment (MacKenzie et al., 2021) [21].

The current qualitative study (phase 2) builds on the initial quantitative results by seeking to develop a deeper and more nuanced understanding of the factors found to promote use of the KT tool during vaccination in phase 1. The integration of quantitative and qualitative methods occurred through connecting the qualitative results to explain the quantitative findings [18], which included revisiting and investigating the domains in the IAM that were identified as influential in phase 1. The aims of this qualitative study were to understand what influenced parents’ perceptions of the relevance of the KT tool and to gain perspective about the influence of contextual factors on parents’ decision to use the tool or not.

## Methods

### Study design

The study was approved by the IWK Health Centre Research Ethics Board. Participants were provided with a copy of the consent form for review via email prior to their participation and additional verbal consent was obtained by the interviewer prior to interview participation. The study followed a qualitative description design. Qualitative description is a qualitative framework that allows the researcher to obtain a concrete yet rich summary of an experience as described by participants in their own words [22]. This design is particularly relevant for understanding phenomena of which little is known [22], and as such, is recommended for research on health care service, given its focus on eliciting concrete responses to questions regarding health care services and the clinical environment [23].

The study design incorporated best practices for patient engagement, a process whereby patients and families are partners in research [24, 25]. In the present study, parents (hereafter referred to as “parent partners”) assisted in development of study materials, including the consent form and interview guides. These materials were piloted with two mothers and one father whose children experience pain related to a health condition. Parent partners were asked to assess the relevance, clarity, and feasibility of the materials. Parent partners were engaged in developing and revising the interview questions and piloted the interview guides by participating in an interview with the principal investigator.

### Participants

**Fig. 1:**
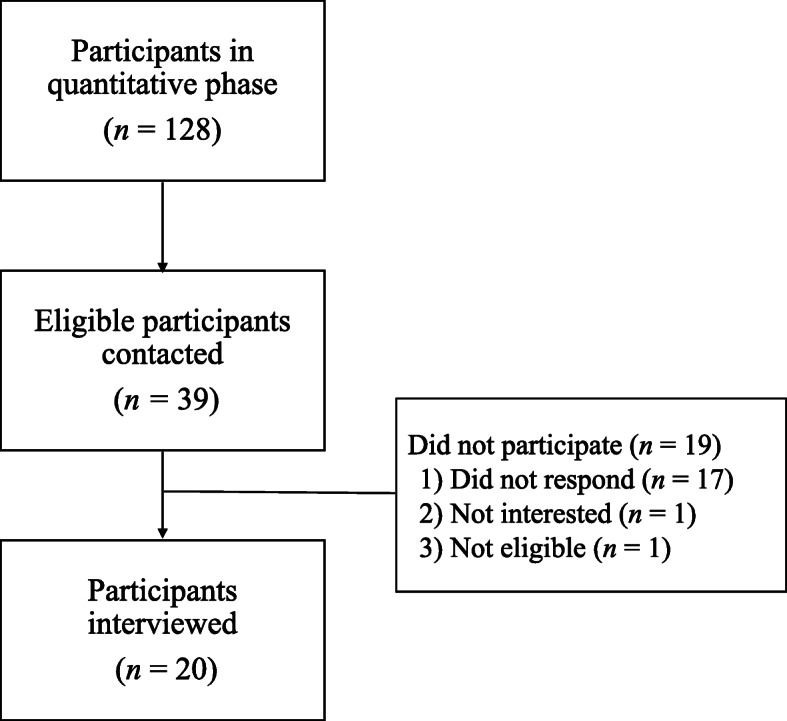
Recruitment Flow Chart

A subset of parents who took part in the quantitative phase of MacKenzie et al.’s study (2021) [21] were recruited for the current study using a maximum variation sampling approach, which is commonly used in qualitative research to target cases of interest related to a specific topic or phenomenon [26]. Parents were purposefully recruited via email to capture parents with a range of characteristics and experiences (e.g., child age, type of strategy used from the KT tool, plans for future strategy use from the KT tool, etc.). Parents who provided consent to be contacted for research in the initial study survey were contacted regarding participation in the current study via email approximately six months following completion of the initial survey. Parents from the overall sample were eligible to participate if they reported that they had vaccinated their child in the time following the initial survey. A total of 39 parents of the 128 who completed the follow-up survey were contacted to participate (see Fig. 1 for recruitment flow chart). The pool of 128 participants from the phase 1 study were recruited using convenience sampling via social media (e.g., Twitter, Facebook), e-newsletters, and website posts. There were a final 20 parents who took part in the interviews (see Table 1 for participant demographics). This sample size was based on established recommendations regarding appropriate sample size for qualitative interview studies addressing research questions of this nature and for the analytic technique utilized (see Data Analysis) [27–30].

**Table 1:** Participant Demographics

	*n* (%)
Age Group	
20–29	3 (15)
30–39	11 (55)
40–49	5 (25)
50–59	1 (5)
Parental Role	
Mother	19 (95)
Father	1 (5)
Highest level of education	
College Diploma	2 (10)
Some University	1 (5)
University Graduate	9 (45)
Graduate Degree/Professional Training	8 (40)
Marital Status	
Married/Common Law	19 (95)
Never Married	1 (5)
Ethnicity	
Asian	1 (5)
Caucasian	19 (95)
Child’s Age Group	
< 1 year	4 (20)
1–2 years	4 (20)
3–4 years	8 (40)
5–8 years	2 (10)
12–15 years	1 (5)
16–17 years	1 (5)

*Note. N* = 20

### Data collection

Parents took part in individual in-depth semi-structured interviews that lasted 32 min on average (range 15 to 48 min). Telephone interviews have been demonstrated to be an effective and feasible method to conduct qualitative research [31] and is particularly advantageous when capturing the perspectives of individuals over a wide geographical area [32], as was the goal in this study. Parents were not exposed to the KT tool again prior to their interview in an effort to capture the aspects of the tool and the experience using it that stood out in parents’ memories following their child’s vaccination. Interview guides (see Additional Files) consisted of open-ended questions and potential probes that were largely informed by the findings of the quantitative phase in the larger research study and that were developed by the study investigators and parent partners. The first author (N.M.) conducted all interviews. Participants were located across Canada and therefore interviews were conducted over the phone. Interviews were recorded and transcribed verbatim.

### Data analysis

The data were analyzed using reflexive thematic analysis with an inductive approach [33]. Reflexive thematic analysis, commonly utilized in qualitative description studies, involves identifying and organizing groups of ideas from interviews into coherent themes to understand shared experiences [34–36].

Following the standard steps for conducting thematic analysis outlined by Braun, Clarke, and Terry [37], investigators developed preliminary codes and assigned data to them using line-by-line coding using data management software (NVivo 12, QSR International). Analysis continued by collating the initial codes into broader, overarching themes. The analysis was led by the first author (N.M.) and at each step, the analysis was reviewed by a second author who is experienced in qualitative methods (P.T.). Rigor and trustworthiness were established by maintaining an audit trail outlining analytic decisions and thick description of findings supported by verbatim quotes from parents, and triangulation of findings by the investigators.

## Results

### Participant characteristics

A total of 20 parents participated in the present study, with children between the ages of 0 and 17 years of age. Of the parents who participated in this study, the vaccinations children received included the influenza vaccine (*n* = 8), a routine childhood vaccination (*n* = 6), a combination of the two (*n* = 5), and a travel vaccination (*n* = 1). The majority of vaccinations took place in a doctor’s office (*n* = 11) followed by a public health clinic (*n* = 5), pharmacy (*n* = 3), and other (*n* = 1). The majority of parents reported using a vaccination pain management strategy (*n* = 18), with the primary strategy being distraction.

### Qualitative results

The analysis generated three interrelated themes that describe factors associated with parents’ use of the evidence-based pain management strategies presented in the KT tool.

#### Theme 1: relevance to parents’ needs and circumstances

A key factor related to parents’ use of evidence-based pain management strategies from the KT tool was how relevant they felt the information in the tool was to information needs they had specifically related to managing their child’s pain during vaccination. This included the degree to which they felt the information addressed gaps in their knowledge or understanding of evidence-based vaccination pain management. This also encompassed the applicability of the information to their children’s various needs (e.g., age, interests, past experiences) and how useful and practical they felt that the information would be.

Parents described feeling that the KT tool was relevant when the information answered a specific question or met a particular need they had within the context of managing their child’s vaccination pain. For instance, parents who expressed wanting to learn how to better manage their child’s vaccination pain described the tool as relevant because it outlined age-appropriate pain management recommendations and provided practical information about how to implement the strategies, such as when to discuss an upcoming vaccination with the child. When parents described the KT tool as relevant to their needs and circumstances, they seemed to be more likely to want to use information to manage their child’s pain. For example, some parents expressed concerns and questions regarding the implementation and safety of some approaches to managing vaccination pain, however others also reported that the tool clarified their misconceptions and expressed that it met their information needs regarding the appropriate use of these strategies.

*… Knowing that you were able to put that [topical anesthetic] cream on there, I didn’t realize that you were allowed to do that. I just kind of assumed that that might have like a drug interaction or something. I was a little nervous to do anything like that. (Mother of a toddler)*

While many parents reported that the tool met their information needs, others discussed how the tool was less relevant as they did not have any information needs regarding managing their child’s vaccination pain.

*It’s interesting to have that information. And I guess, yeah, I just didn’t think that I needed it at that time. (Mother of an infant)*

Similarly*,* parents described the importance of the applicability and practicality of the information in the tool to their circumstances as a facet of relevance. Parents described that the information in the tool was relevant because it provided a range of pain management strategies that they felt were diverse in type (i.e., pharmacological or non-pharmacological), feasibility, and suitability for children of varying ages. This seemed to give parents a sense of reassurance that if it was not possible to use one particular strategy, they could make another choice which was better suited for them and their child. For example, parents described that even if they were unwilling or unable to use one particular strategy, they felt that the tool still had relevance as it provided alternative options that they were more comfortable using.

*… Am I making the right choice? And then what other tools do I have? Because you’re still learning how to breastfeed … So things that you would typically use to comfort your child … you might not be comfortable enough to utilize that in the moment. So [it is helpful] to have another list of 10 things that you can do as a parent. (Mother of an infant)*

The relationship between the relevance of the tool and parents’ use of the evidence-based information seemed to not only be about meeting parents’ information needs with new content, but also validating the strategies parents were already using. When parents recognized familiar or previously used strategies in the KT tool, they described feeling validated in their choice and seemed to view the tool as more relevant for their own needs as well as their child’s. Identifying familiar strategies seemed to strengthen the relevance of the tool and the likelihood that parents would continue to use evidence-based pain management strategies for their child’s vaccinations in the future. Thus, the KT tool appeared to be perceived as relevant as it reflected parents’ past pain management behaviours or plans for the future.

*… It was reaffirming like yes, these are things that work, instead of me just being like, ah, I guess it might work. Like it gave me information that just sort of validated that what I was choosing to do like actually had merit and it wasn’t just ‘I think this is going to work.’ It wasn’t just a hypothesis, it was like this has been proven with other people. (Mother of a toddler)*

#### Theme 2: alignment with personal values and experiences

Another factor integral to parents’ use of evidence-based strategies from the tool was the degree to which the information in the KT tool fit with parents’ values related to vaccination pain management. Parents’ values in various domains, such as how important it was to them to integrate credible and reputable information into decisions about their child’s health, as well as the importance they placed on vaccination pain management, appeared to play a key role in determining whether parents planned to use the strategies presented in the KT tool.

Many parents described the personal value placed on the importance of finding and using credible and trustworthy information when making decisions related to their child’s health. Parents reported feeling confident in the tool because they knew that the pain management strategies presented were backed by research, that the information was vetted by experts in the field, and that the tool was associated with reputable organizations known for research in this area (e.g., by seeing the logos on the tool). For these parents, having this value met and knowing that the tool presented the best possible pain management information for their child’s vaccinations made them feel confident in engaging with the tool and ultimately led to a sense of empowerment.

*I felt confident that [the tool] would have been developed in a way that I didn’t have to look any further … .I wouldn't have to go any further to know that they had distilled out what I needed to know to feel confident in trying some strategies. (Mother of an infant)*

However, for other parents, managing their child’s pain during vaccinations was not something that seemed to fit with their core values and beliefs. For instance, some parents reported feeling that vaccination pain is not intense or long enough to warrant management. Parents who placed less of a priority on vaccination pain management and did not express worry about their child’s experience of getting a vaccination expressed that they were less likely to utilize the evidence-based information presented.

*I don't think that vaccines are really that big of a deal. You know, in terms of pain, I mean it’s a bit uncomfortable for a split second but then it goes away. So it wouldn't be something that I would be like kind of anxious about and like “how am I going to relieve my child’s stress” because I personally myself don’t find them that stressful. (Mother of an infant)*

Similarly, not all parents agreed with the principles of the evidence-based strategies themselves and expressed preferences in how they would manage their child’s vaccination pain which contrasted with some strategies presented in the tool. For example, some parents preferred not to use pharmacological strategies, while others felt that using any strategies at all would be excessive given their child’s needs.

*I’d probably be more inclined to use natural ones like nursing and things like that, less inclined for distraction just because of my parenting style, or using an EMLA patch. (Mother of an infant)*

#### Theme 3: support of the clinical environment

Beyond the information itself, a central factor related to parents’ use of the evidence-based strategies presented in the tool was the level of support they received from the clinical environment. Clinical support of parents’ desire to use evidence-based pain management strategies took many forms, from the physical layout of the environment, to their interactions with the HCP. Both the physical environment and interactions with the HCP set the tone for parents’ experiences utilizing the KT tool during the vaccination appointment.

The physical environment was a prominent facilitator of parents’ use of the evidence-based pain management strategies during their child’s vaccination. Many parents reported feeling at ease when the clinical environment encouraged strategy use. This included signage (e.g., “breastfeeding is encouraged”), private exam rooms, and availability of resources to facilitate using the tool (e.g., having bubbles and toys available for distraction).

*… [The vaccination] is all in a nice little private room that had the rocking chair and it had the nursing pillows and it had books for the kids, and stuff like that. So I think it was a better physical environment. (Mother of a school age child)*

Interactions with the HCP were also integral to parents’ use of the evidence-based strategies during their child’s vaccination. When HCPs validated and encouraged parents’ use of pain management strategies, parents reported feeling more confident in using the evidence-based techniques to manage their child’s pain. Parents reported that when they had positive interactions with HCPs around vaccination pain management, it is easier to implement the strategies, and it may even facilitate collaborative conversations about how a given strategy could be best used (e.g., how best to position the child) or by having the HCP co-facilitate use of a selected strategy (e.g., aiding with distraction).

*… I know during some of the vaccines, [the physician] had a rattle that she’ll distract the child with. So maybe if I’m not using the strategy, my physician is using the strategy. (Mother of an infant)*

Furthermore, when HCPs themselves raised the topic of strategy use, parents interpreted it as normalizing their desire to implement strategies and felt more comfortable talking about how they wanted to use the tool. When parents felt supported by their HCP during these interactions and it ultimately helped parents feel that their use of the tool was welcome.

*I think kind of the more [strategy use] becomes the norm, the more parents that know about it and adopt these strategies. I’m interested and kind of encouraged to see a bit of that culture change in the next while where this can just kind of become the norm and you don’t feel like you’re just a weird parent walking in with all the bubbles and everything but it's just kind of accepted practice. (Mother of a toddler)*

Conversely, many parents reported experiences where they did not perceive that their HCP supported their use of the evidence-based strategies from the tool. These parents described feeling apprehensive about using the pain management strategies for their child’s vaccination for fear of challenging the HCP’s authority or disrupting the overall vaccination procedure. In some cases, parents described feeling concerned that their child’s HCP’s may view them negatively for wanting to implement pain management strategies for a vaccination.

*I don't want my doctor to think that I’m some crazy person … I don't want my doctor to think that I’m like that because if I come in with something else, they might think, ‘Oh, there's that mom, she’s worried about everything.’ They might not take me as seriously with other things. (Mother of a toddler)*

Other parents described a perceived power differential that posed a barrier to talking about using the tool with their HCP, despite knowing they wanted to use the evidence.

*I might actually feel a little bit uncomfortable trying to like show [the HCP] this even though, you know, I believe it and I know it to be true … We didn’t want to like feel like we were being disrespectful to what the doctors and the nurses were trying to do. (Father of a toddler)*

While clinical support was a key factor in supporting parents’ use of evidence-based pain management strategies, a few parents who felt very strongly about the importance of pain management described assuming an advocacy role for their child, by going ahead and using the pain management strategy of choice regardless of their HCP’s opinion.

*… If the unsupportive healthcare practitioner hadn’t been supportive, that’s tough, I’m still going to do what I think is best for my kid. Pain management is kind of important. (Mother of a toddler)*

## Discussion

The present study identified three themes which provide a deeper understanding and contextualization of the factors found to promote use of the KT tool during vaccination. These included the relevance of the tool to parents’ needs, the degree to which the tool aligned with parents’ values, as well as the support parents received from the clinical environment. The exploration of these needs and values, as well as understanding what aspects of the KT tool, and experience using it, influenced parents’ perception of its relevance to their context and needs, is important to provide further context to understanding what promotes use of such tools.

### The implications of relevance

Ensuring KT tools are relevant to parents’ information needs, as well as their past experiences, is a key component of effective dissemination. Specifically, considering the context in which information will inform knowledge, decision-making, and behaviour is related to the relevance of the information in the given situation, and is determined by the knowledge user themselves [38]. These concepts are consistent with the domain of relevance within the IAM framework. Without consideration for the relevance of the evidence shared with the intended knowledge user in the development of KT tools, goals such as changing attitudes and beliefs in favour of engaging in a certain behaviour are more difficult to achieve [38].

Relevance of the KT tool to parents’ needs and circumstances took many forms in the present context, including filling knowledge gaps and clarifying existing misconceptions about some pain management strategies. Several parents described the KT tool as having contextual relevance to them. This led to a sense of affirmation in vaccine-accepting parents’ sense of what they could do to support their child’s vaccination pain management and also reinforced past strategy use. Therefore, although many parents were already using the effective pain management strategies presented in the KT tool prior to viewing it, the tool served to reinforce parents’ strategy use and build their confidence regarding their benefit for their child. This conclusion is consistent with evidence demonstrating that mothers who breastfed for pain management during their infants’ needle procedures more frequently used that strategy at future vaccination appointments when they also saw it presented in a KT tool [17]. Thus, seeing strategies which reflect past pain management behaviour can relate to the perceived relevance of the KT tool.

Parent-reported relevance of the KT tool pertained to the range of evidence-based pain management strategies from which parents could choose*.* Evidence-based resources that present a broad range of information have been shown to facilitate parents’ sense of feeling reassured and being in control of their child’s health, which in turn correlates with intentions to use the information [39]. Another consideration related to the importance of having a broad range of strategies is cultural and personal factors. For example, strategies such as breastfeeding may not be ideal for some parents to utilize due to personal and cultural preferences around breastfeeding in public, or if the child is accompanied by a caregiver who cannot breastfeed. Therefore, socio-cultural diversity issues are important to attend to in the creation of KT tools, as the type of information individuals are looking for, what is considered relevant, and how trustworthy the information is may differ cross-culturally based on their cultural norms and preferences [40]. Thus, tools such as the one studied here may have greater relevance when parents have a range of strategies to choose from that relate to their needs, as they can find alternative strategies to use within the same resource.

Engaging in patient-oriented research could thus be central to the KT process to ensure tools are relevant to knowledge users and ultimately used. Patient engagement is important to ensure relevant questions are asked and that findings are tailored to knowledge users [41, 42]. In this context, parent engagement could help researchers to identify strategies of interest to parents regarding vaccination and could shed light on misconceptions to address in a tool such as this.

### The role of values in KT tool uptake

When parents’ values of managing children’s vaccination pain aligned with their value of information credibility, they felt reassured using the information. Parents’ recognition of the tool as credible created a sense of trust leading to reassurance in tool use. This strongly aligns with existing literature, where parents not only report that credibility in an tool is important, but that it increases their trust in the information they are reading [43]. It is important to note that trust in such tools to share health information may differ across socio-cultural groups, including trust of the source of the information [44]. Ultimately, parents’ trust in the tool related to their confidence in strategy use, a common theme for uptake of KT tools [9], as many discussed trusting they were doing the right thing for their child. This was empowering for parents as they could make informed choices to manage their children’s vaccination pain, use evidence, and be an active participant in their child’s healthcare as opposed to simply being a passive observer.

### Nature of interactions with health care providers

Interactions with HCP can influence uptake of the tool and can make parents feel supported and validated in their use of a KT tool or can make them feel apprehensive about using a tool at all. The support from the HCP was of particular importance and was noted as something parents related to their confidence in tool use. The desire for support from the HCP is not uncommon as many parents check in about strategy use with their HCP prior to a vaccination and can lead to opportunities to discuss pain management [43, 45]. HCPs validating parents’ management of vaccination pain can reinforce tool use and further parents’ trust which can be fundamental to parents’ integrating health information into decision making and behaviour [46].

When HCPs did validate parents, and engaged in positive dialogues regarding tool use, parents perceived feeling very supported and encouraged. This validation was impactful to the point where several months later (i.e., at the time of the interview), parents were still reflecting on how important it was for them to feel that they had the support of the HCP in the decisions they were making for their children. Overall, it is clear parents are signaling that they do not have grand expectations from their HCP to support their use of KT tools in the clinical environment. Rather, simple statements validating parents’ choices and offering support (e.g., help with positioning) appeared to make a significant difference in the degree to which parents feel supported by their HCPs. This was especially impactful when parents perceived that the physical environment facilitated their strategy use.

Conversely, when parents had negative or challenging interactions with their HCP, who did not support their use of the tool, this led parents to react in one of two ways. Parents either persevered, taking on the role of an “advocate” for their child (i.e., insisting that they would go ahead and use the tool despite what the HCP thought) or became apprehensive, not wanting to challenge the authority of the HCP and subsequently not using the tool. Thus, parents’ perceptions of unsupportive reactions from HCPs can influence how parents engage with KT tools. Prior work has shown that in cases where HCPs have denied parents’ plans to use pain management strategies during vaccination, this was often because HCPs were not aware of the evidence and parents did not insist on using the strategies [17]. Therefore, it is important that HCPs also be involved in KT activities [17] to ensure they are familiar with best practices which can subsequently support parents’ uptake of evidence. Further to this, it is important to understand the barriers that HCPs face (e.g., time, education, etc.) when implementing KT tools and other interventions [47], as well as identifying those barriers which are most important (and possible) to change [48].

### Strengths and limitations

This qualitative research is unique in that it builds upon rich, two-phased quantitative data that identified core factors shown to be impactful in promoting the uptake of evidence-based practices presented in a KT tool (MacKenzie et al., 2021) [21]. The inclusion of the findings from the prior quantitative study phase informed the current study design, particularly in the development of the research questions and interview guides. In using this approach, the current study was able to qualify what these important variables such as “relevance” and “confidence” actually mean to parents in a clinical context, which provides valuable insight into how these factors can be better addressed in KT tool development.

Interviews with parents took place, on average, six months following their child’s vaccination, and while the majority of parents appeared to have very clear recall of their experiences, this delay presents the risk of recall bias. Sooner follow-up may have facilitated even more reliable and informative conversations. As well, while telephone interviews have been shown to be an effective and feasible data collection method [31], the inability to view body cues or other nonverbal cues may limit the context of the information communicated [32]. It is also acknowledged that these experiences are reflective of a small subset of parents (predominantly mothers) with minimal ethnic and socioeconomic diversity. Thus, it could be that feelings of power imbalances between the parent and HCP that hindered KT tool use may be underestimated in the present sample, whereas this experience may be exceptionally salient when greater degrees of marginalization or oppression are present in the parent’s sociocultural context. Intersecting identities related to socio-economic status, race, gender, or health status have been demonstrated to significantly impact health care and outcomes [49]. The pressure to adhere to what their HCP recommends to avoid being labeled a ‘problematic patient’ [50] is known to be experienced by families from racialized and less privileged populations to an even greater degree [51]. Therefore, these cultural positions and identities of both patients and HCPs may influence engagement around KT tool use, and should be explored in future research reflecting a broader representation of diverse participants. Relatedly, it is noted that this sample included a single participant who identified as a father. Fathers and mothers differ in their approaches to children’s pain management, both in terms of behavioural approaches and in pharmacological management of pain [52, 53]. Thus, fathers may value different strategies or approaches over those primarily endorsed by mothers. Furthermore, research has shown that fathers see pain management as a lower priority relative to mothers [54] and are more likely to believe that coping with pain independently is something children should learn [55]. As such, it is possible that fathers may place different priorities on different aspects of pain management, which in turn, may influence how they utilize a resource such as the present KT tool. However, given our sample composition, the current data cannot speak directly to this issue. Another limitation is the possibility that participants in this study, all of whom had vaccinated their children, may be more inclined to engage in health-seeking behaviours, which could positively bias their impressions of the experience using the KT tool and the strategies it presented.

### Future directions

Future research should explore specific socio-cultural factors implicated in parents’ use of KT tools. Ensuring broad representation of diverse individuals in future research is an important step toward deepening the understanding of how best to disseminate and promote evidence about pediatric pain and broader health to parents and other knowledge users. Furthermore, given the further understanding of factors related to parents’ uptake of KT tools in this study, future research should explore institutional and policy level changes that could facilitate effective dissemination of evidence to parents and mitigate any identified barriers to their uptake.

### Implications and conclusion

The present study uncovered what makes a KT tool relevant to, and considerate of, parents’ values and how this relates to confidence and tool use during a child’s vaccination. These findings can inform the development of future KT tools to consider what is relevant to a given knowledge user and subsequently integrate this into tools.

As KT activities become increasingly common as primary research objectives, the importance of addressing uptake of these interventions, such as KT tools, also increases. For KT tools geared toward parents, ensuring relevance and a values match will also become increasingly important. With additional support from an HCP, parents can feel confident and empowered in their ability to support their children during painful procedures, ultimately promoting higher quality care and pain management for children through evidence uptake.

## Supplementary Information

**Additional file 1.** KT Tool.

**Additional file 2.** Semi-Structured Interview Guide.

## Data Availability

The datasets used and/or analyzed during the current study are available from the corresponding author on reasonable request.

## Abbreviations

KT: Knowledge Translation
HCP: Health Care Provider
IAM: Information Assessment Method
