# Supplementary material for: Factors associated with parents’ experiences using a knowledge translation tool for vaccination pain management: a qualitative study
Source: BMC Health Serv Res. 2021 Apr 16;21:355. doi: 10.1186/s12913-021-06326-2 (PMC8052692; doi:10.1186/s12913-021-06326-2)
Supplement: Supplementary file 1 — Additional file 1. KT Tool. [file 12913_2021_6326_MOESM1_ESM.pdf]

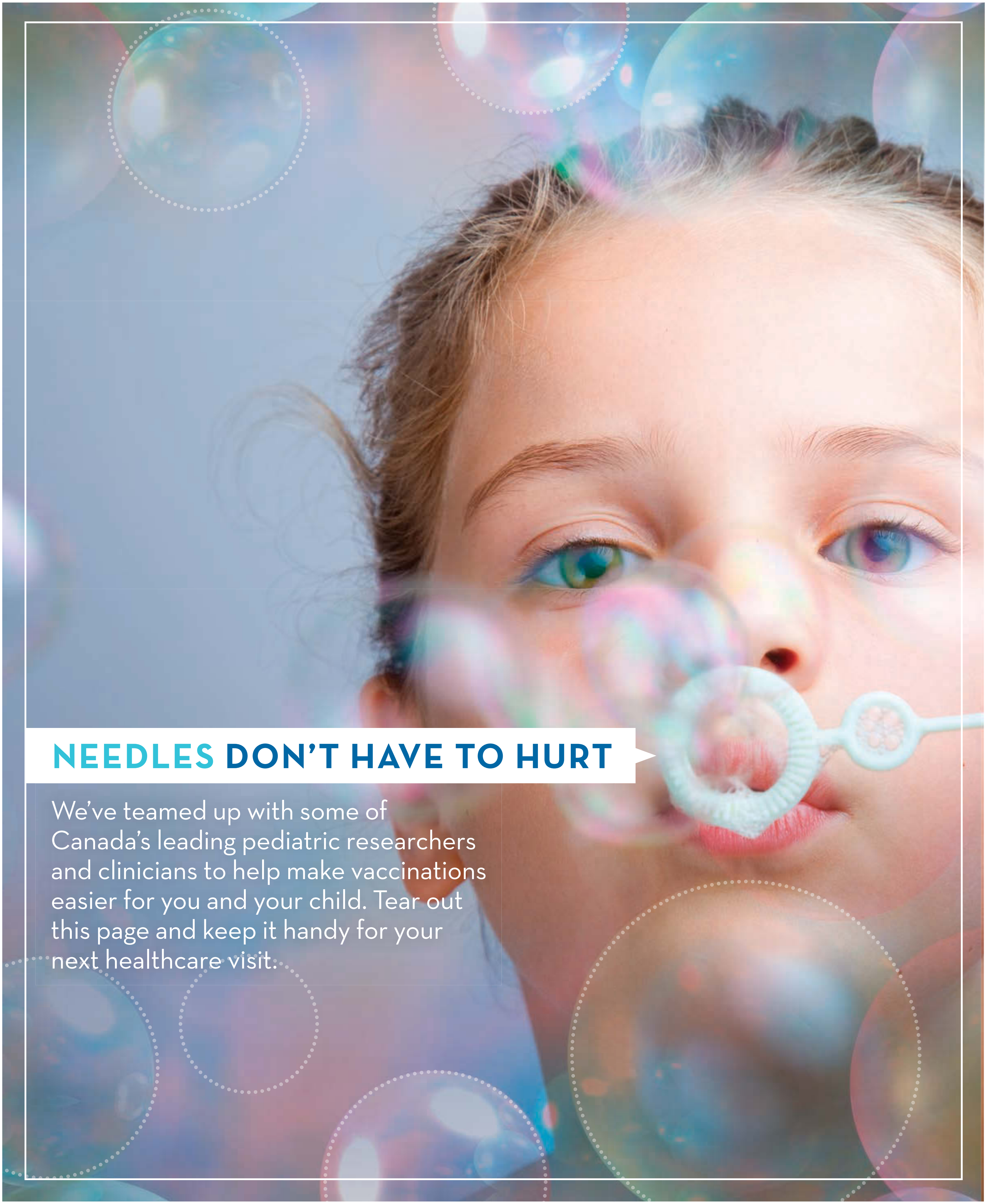
NEEDLES DON'T HAVE TO HURT
We've teamed up with some of Canada's leading pediatric researchers and clinicians to help make vaccinations easier for you and your child. Tear out this page and keep it handy for your next healthcare visit.

NEEDLES DON'T HAVE TO HURT

Keep this timeline handy to make your child's vaccinations easy peasy

A FEW DAYS BEFORE THE NEEDLE

ALL AGES

- **Be honest.** Talk with your children about strategies to manage their pain and distress.
- Ask children over 10 if they want **you to be there for the needle**.

30-60 MINUTES BEFORE THE NEEDLE

- Apply **numbing creams or patches** (topical anesthetics such as EMLA™, AMETOP™, and Maxilene™), available for purchase from pharmacies without a prescription. Follow instructions – product must be applied 30 to 60 minutes before the needle to the area where the needle will go.
- Apply cream to multiple areas if your child is having more than one needle.
- Ask if you are not sure exactly where on the body the needle will be given.

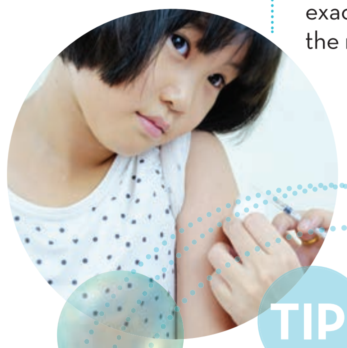

TIP:

IF MORE THAN ONE NEEDLE IS TO BE GIVEN AT AN APPOINTMENT, ASK THE HEALTHCARE PROVIDER TO GIVE THE MOST PAINFUL VACCINATION LAST.

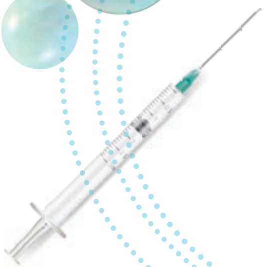

RIGHT BEFORE, DURING AND AFTER THE NEEDLE

ALL AGES

- **Stay calm** and interact normally with your child.

BABIES UP TO AGE TWO

- **Sit upright.** Newborns (younger than one month) can be held skin to skin against your chest. Young children can sit on your lap. Hug but don't hold too firmly.

  Needle location:
  - Babies under 1 year: upper outer thigh.
  - Babies 12 months and older: upper arm.
- **Breastfeed** (if possible). If your child unlatches, gently reposition when your child is ready.
  - Or **give sugar water** 1-2 minutes before the needle (1 tsp white sugar dissolved into 2 tsp boiled or distilled water).
  - Or have your child **suck a finger or a pacifier.**

CHILDREN TWO TO 17

- **Use neutral language** rather than drawing attention to pain.
  - Let children/teens know when things are going to start by saying "Ready?" or "Here we go!"
  - Avoid reassuring (e.g., "It'll be over soon" and "You're OK") as it can increase distress and pain.
- **Distract** by taking their attention away from the pain.
  - Watch a video together and ask questions about it.
  - Encourage listening to music through headphones.
  - Talk about something fun (e.g., birthday party).
  - Play with toys that encourage deep breathing, such as blowing a pinwheel or bubbles.
- **Get support** from a registered psychologist if your child has severe needle fear or phobia.

CHILDREN SEVEN TO 17 WHO FEEL FAINT WHEN GETTING NEEDLES

- A simple technique called **muscle tension** can raise blood pressure and stop these feelings.
  - Ask your child to recline or lie down if possible and tighten his/her leg and stomach muscles (not the arm where the needle is going to be given).
  - Tensing should continue for about 20 seconds until your child is feeling flush in the face. Stop tensing for five seconds (without fully relaxing) before tensing again.

BROUGHT TO YOU BY: HELP Eliminate Pain in Kids & Adults; It Doesn't Have to Hurt – Proven Pain Control for Children

SUPPORTER: Immunize Canada / Immunisation Canada – immunize.ca

INSTITUTIONS: IWK Health Centre; SickKids

FUNDERS: Nova Scotia Health Research Foundation; CIHR IRSC – Canadian Institutes of Health Research / Instituts de recherche en santé du Canada

Evidence summarized by Dr. Christine Chambers, Dr. Meghan McMurtry, Dr. Noni MacDonald, Dr. Melanie Barwick, Dr. Anna Taddio, Kathryn Birnie and Katelynn Boerner

Adapted from Taddio A., McMurtry CM., Shah V., et al. Reducing pain during vaccine injections: clinical practice guideline. CMAJ 2015;187:975-982.
