# Supplementary material for: Factors associated with parents’ experiences using a knowledge translation tool for vaccination pain management: a qualitative study
Source: BMC Health Serv Res. 2021 Apr 16;21:355. doi: 10.1186/s12913-021-06326-2 (PMC8052692; doi:10.1186/s12913-021-06326-2)
Supplement: Supplementary file 2 — Additional file 2. Semi-Structured Interview Guide. [file 12913_2021_6326_MOESM2_ESM.docx]

**Semi-Structured Interview Guide**

**Q1.** Have you attended a vaccination appointment with your child since completing our survey on the information sheet on vaccination pain management for kids in the Fall of 2018?

This includes, but is not limited to, routine childhood and/or school-age vaccines and boosters (e.g., DTaP, MMRV, HPV, HepB, Meningococal, etc.) and the flu shot.

**Q2**. How many of your children have you accompanied to a vaccination appointment since completing our survey in the Fall?

***For parents with one child****:* We would like you to consider the pain experiences of your child to answer the following questions.

***For parents with multiple children****:* We would like you to consider the pain experiences of one of your children to answer the following questions. Based on your response to the first question, we have randomly chosen your __________ child *[1^st^ born, 2^nd^ born, 3^rd^ born, etc, insert the birth order number of the child selected]* as the child to discuss.

**Q3.** What type of vaccination did your child receive?

- Routine childhood and/or school-age vaccines and boosters (e.g., DTaP, MMRV, HPV, HepB, Meningococal, etc.)
- Influenza vaccination (e.g., flu shot)

**Q4.** When was your child’s last vaccination appointment (approximately)?

**Q5.** Where did your child’s last vaccination appointment take place? (e.g., doctor’s office, flu shot clinic, pharmacy, hospital, etc.)

**Q6**. Tell me about your child’s vaccination.

- - What was challenging or went well with your child’s vaccination?

**Q7**. What was your impression of the information sheet with the pain management strategies?

- - Did you feel like you could trust the strategies?

**Q8**. Tell me about why you decided to use/not use the strategies.

- - What was your impression of the strategy you chose?

**Q9**. A) [Used strategy] Tell me about some things that helped you implement/make sense of how to use the strategies to manage your child’s pain. Did you face any challenges in using the strategies?

B) [No strategy] What might have made you more likely to use the strategies to manage your child’s pain? Was there anything specific you found difficult/unappealing?

**Q10**. Tell me about what kind of support you had in helping your child manage with the vaccination pain.

- - Did you have family/friend support? Health care provider support?

**Q11**. Do you think the outcomes of your child’s vaccination pain would have been different if you had/had not used the strategies?

**Q12**. Overall, would you change anything about the strategy resource sheet?

**Q13**. A) [Used strategy] Would you use these strategies again in the future?

B) [No strategy] Would you reconsider using the strategies in the future?

**Q14**. In your opinion, how could accessing research-based health information for your children be

made easier?

**Q15**. Is there anything else you’d like to share about your experience managing your child’s pain during their recent vaccination?
